# Motives and Barriers for Regular Physical Activity among Medical Students from the Western Balkans (South-East Europe Region)

**DOI:** 10.3390/ijerph192316240

**Published:** 2022-12-04

**Authors:** Miloš Ilić, Huiwen Pang, Tomislav Vlaški, Maja Grujičić, Budimka Novaković

**Affiliations:** 1 Department of Pharmacy, Faculty of Medicine, University of Novi Sad, Hajduk Veljkova 3, 21000 Novi Sad, Serbia; 2 Australian Institute for Bioengineering and Nanotechnology, The University of Queensland, Brisbane, QLD 4072, Australia; 3 School of Biomedical Sciences, Faculty of Medicine, The University of Queensland, Brisbane, QLD 4072, Australia; 4 Medical Faculty Heidelberg, University of Heidelberg, Im Neuenheimer Feld 672, 69120 Heidelberg, Germany; 5 Department of General Education Subjects, Faculty of Medicine, University of Novi Sad, Hajduk Veljkova 3, 21000 Novi Sad, Serbia

**Keywords:** physical activity, motives, barriers, medical students

## Abstract

Regular physical activity (PA) has multiple beneficial effects on students’ health, effectively reducing the risk of various non-communicable diseases. Various factors play a role in an individual’s motivation to engage in and maintain regular PA. So far, no research dealing with the motives and barriers for regular PA among medical students has been conducted in the countries of the Western Balkans. The aim of this study was to identify the motives and barriers related to regular PA and compare them with different demographic, socioeconomic, and individual lifestyle factors among Western Balkans medical students. The research was conducted in a form of a cross-sectional study. It included 2452 medical students from 14 faculties in five countries (Slovenia, Croatia, Bosnia and Herzegovina, North Macedonia, and Serbia). The most commonly reported motive of medical students for regular PA is to feel better, followed by stress reduction, to look better, the desire to lose weight, and to control chronic disease. PA improvement motives are more frequently associated with the female gender, more advanced years of study, a normal weight, above average household income, and a non-smoking status. Faculty obligations are the most common barrier for regular PA among medical students, followed by other barriers, such as financial situation, current life situation, and health conditions. Barriers are more frequently reported by male students who are overweight or obese, who have a below average income, and are smokers. It is necessary for public health authorities to examine and take into account the perceived motives and barriers when forming activities and policies that aim at increasing the level of PA, in order to enhance the health of the student population.

## 1. Introduction

Physical activity (PA) is recognized as one of the major determinants of promoting well-being and enhancing overall health [1]. Regular PA has a significant impact on all human organ systems, mediating multiple beneficial effects on the overall health, effectively reducing the risk of various noncommunicable diseases (NCD), such as insulin resistance, diabetes, dyslipidemia, hypertension, and cardiovascular diseases [2,3].

Healthy life habits (proper nutrition and regular PA) are essential for the cognitive and social development of young adults and can play a key role in their overall academic success [4].

Students represent a specifically vulnerable group, since they tend to change their habits and nutritive patterns upon enrolling into universities [5]. Due to the requirements of faculty courses, students are often physically inactive for prolonged periods during the day and are prone to consuming unhealthy meals (high energy, rich in sugars, unsaturated fats, and salt), which can consecutively have negative health consequences [6,7].

Insufficient PA of young adults is one of the most obtrusive current challenges of public health [8]. Global research estimate that 20–50% of young adults engage in an insufficient amount of PA [9,10,11,12].

There is a plethora of factors that are negatively associated with lower PA level, such as the female gender, a lower socioeconomic status, rural residential community, lower level of education and a less developed health system, alcohol and tobacco consumption, bad social influence, as well as individual factors (perceived motives and barriers) [9,11].

Motivation can be defined as a process of initiating and directing activity toward reaching a goal that satisfies a specific need [13,14]. The term “motive” or “motivator”, for PA, is applied mainly to an inner urge (reason) that prompts a person to do regular PA [13,14].

Some of the most common motives are improving one’s health, improving mood, and enhancing one’s physical appearance [15]. Motives for practicing regular PA can be different, in relation to gender [15,16]. Some of the main motivators towards PA for men are the improvement of physical health and appearance, while for women, in most cases, it tends to be weight loss and a boost in self-confidence [15,16]. It is regarded that a range of factors affects the individuals’ motives for PA, including personal (demographic, biological, and psychological), environmental (related to the social environment and the physical environment), and behavioral [17]. The level of education, existing lifestyle habits (such as smoking and alcohol consumption), as well as health conditions, also play an important role in the motivation for PA [17].

Barriers could be defined as objective or subjective reasons that can prevent a person from completing a defined task and are tended to be more similar among different age groups [18]. Some of the most common barriers for adults are a lack of free time and a bad financial situation [16,18,19]. When it comes to the student population, similarly, one of the most frequent barriers toward having regular PA is lack of time, due to faculty obligations, as well as a lack of money to pay for memberships to fitness centers [16,18,19].

Given the importance of various factors and the role they play in an individual’s motivation to engage in and maintain regular PA, it is essential to understand how motivators and barriers influence PA, in such a complex and multidimensional environment [13,14,15,16,17,18,19]. Understanding the relationship between personal, environmental, and behavioral factors, with specific activity-related factors, is crucial for developing appropriate programs and interventions, in order to increase PA [13,14,15,16,17,18,19].

Medical students represent a group of particular interest, in terms of lifestyle habits and PA [20]. Several studies had observed that medical students have higher odds of experiencing burnout symptoms during their studies, in relation to other students [20]. PA is seen as one of the factors that can increase stress resilience and coping with cognitive and emotional burdens, and enhance the overall well-being of students [21].

As future healthcare workers, they are responsible for promoting and advertising a healthy lifestyle among the general population [22]. One of the proposed arguments is that patients tend to disregard lifestyle advice from those health professionals who do not practice that advice themselves [22]. This denotes the importance of establishing healthy lifestyle habits during their professional formation [22].

So far, no research has examined the motives and barriers for regular PA, among the medical students in the Western Balkan countries (Slovenia, Croatia, Bosnia and Herzegovina, North Macedonia, and Serbia).

The aim of this study was to identify the motives and barriers related to regular PA and compare them across the different demographic, socioeconomic, and lifestyle-related factors among Western Balkan medical students.

## 2. Materials and Methods

### 2.1. Study Design and Population

A cross-sectional study that included 2452 students from 14 medical faculties in the Western Balkans, was performed from November 2019 to February 2020. A convenience sampling of faculties of medicine was used. The participating medical faculties from each county were shown in Table 1. Female students made up 2015 (82.2%) and male students 437 (17.8%) of the sample.

### 2.2. Study Questionnaire

An online Google form survey was used as an instrument for data gathering. Accessible from any device, the survey was distributed using channels, such as emails, social networks (Facebook), or faculty websites. Participation was voluntary and participants were allowed to withdraw at any time. Only fully completed questionnaires were registered in a database and downloaded as a Microsoft Excel sheet, upon a study completion. The research method through Google’s privacy policy guaranteed the anonymity of the respondents.

### 2.3. Variables

The survey was divided in two parts. The first part contained the questions about the general characteristics of the respondents (attended faculty, gender, year of study, body height and weight, household income, type of settlement), and the second part contained the questions about life habits (daily level of PA, alcohol consumption and student smoking status), and questions about reasons (motives) for engaging in regular PA, and excuses (barriers) for irregular PA.

Most common motivators (motives) were determined with the statements: “I engage in regular PA because I want: to feel better (mood improvement, energy boost, better sleep, fun, etc.), to reduce stress (relaxation and improving executive functions, e.g., thinking, learning, and judgment skills—to improve academic performance), to look better (better physical appearance), to lose weight (reduce adiposity), to control chronic disease (manage chronic conditions and disabilities), and I do not engage in PA.”

The perceived barriers for engaging in regular PA were determined with the statement: “I do not engage in regular physical activity due to: faculty obligations (lack of time), current life situation (special events, day-to-day or global circumstances, or personal circumstances that affect the person or their family or friends, e.g., illness or a situation that requires care), financial situation (e.g., high costs of equipment or access to sports centers), health conditions (injury, illness, chronic medical condition, etc.), other barriers (subjective or objective), and I engage in PA.” Both statements were multiple-choice type, so respondents could select one or more offered answers.

Based on daily regular PA, students were divided according to those who engaged in some kind of regular PA and those who did not.

Other independent variables are analyzed as follows:

Nutritional status was determined by calculating the body mass index (BMI), dividing the self-reported body weight (kg) and body height squared (m). Based on the obtained values, and using the WHO recommendations, the participants were classified as: underweight (<18.50 kg/m^2^), normal weight (18.50–24.99 kg/m^2^), overweight (25.0–29.99 kg/m^2^), and obese (≥30.0 kg/m^2^).

The groupings according to the years of study (1–6), two categories were defined: first-third year of study and fourth-sixth year of study.

Students were divided in three groups by self-evaluated household income: below average (far below and below), average, and above average (above and far above).

Based on the number of inhabitants, the respondents were classified according to the type of settlement lived in before starting university education, and two categories were determined: rural and urban.

Alcohol consumption was determined by the self-reported answers, according to the students who do not drink, those who drink occasionally, or on weekends, students who consume alcohol several times a week, and daily drinkers.

Students were grouped, based on their smoking status, as “smokers” (for occasionally smoking or any other number of cigarettes smoked per day), and “non-smokers” (I am not a smoker).

### 2.4. Statistical Analysis

In the statistical analysis, the categorical variables were displayed as numbers and percentages. The association between the independent variables and the motives/barriers were examined using the χ^2^ test. As a measure of association, the Fi coefficient (for 2 × 2 tables) and Cramer’s V (for tables higher than 2 × 2), were used.

The relationship between the outcome (dependent) variable, i.e., the engagement in some form of regular PA and the motives/barriers, was tested using a multinomial logistic regression (MNLR).

In the first step, the univariate MNLR was conducted by examining the association of only one independent with the outcome variable, whereby “has engaged in some form of regular PA” was taken as the reference category. Those variables that showed a statistically significant association with the outcome variable were controlled for the possibility of other independent variables (multivariate MNLR).

Spearman’s correlation coefficient was used, in order to examine the corelation between the two numeric variables.

SPSS Statistics for Windows version 24 (IBM Corporation) was used for the statistical analysis. The statistical significance was taken into account for the *p* values lower than 0.05.

### 2.5. Ethical Aspects of the Research

The study was conducted, according to the guidelines of the Declaration of Helsinki. The Ethics Committees/Commissions of the faculties that participated in the research gave an opinion that approval of the Committees/Commissions was not required, as the research did not include invasive methods and did not violate the privacy of the respondents.

## 3. Results

Medical faculties differed significantly in gender distribution (χ^2^ = 50.032, *p* < 0.001, fi = 0.143) with the highest presence of female students (91.1%) at the Faculty of Medicine of the University of Rijeka, Republic of Croatia, and the lowest (73.7%) at the Faculty of Medicine of the University of Zenica, Bosnia and Herzegovina, compared to other faculties (not shown in tables).

Regardless of the attended faculty, most students engaged in some kind of regular PA (62.3%) (not shown in tables). Engagement in some kind of regular PA significantly differed between medical faculties (χ^2^ = 78.203, *p* < 0.001, fi = 0.179) (Table 2). The students of the Faculty of Pharmacy and Biochemistry of the University of Zagreb, Republic of Croatia, in the smallest percentage, engaged in some kind of regular PA (49.1%), while the students of the Faculty of Pharmacy of the University of Mostar, Bosnia and Herzegovina, were the most physically active, in comparison to students of other faculties (77.3%).

Male students engaged in some form of PA significantly more often (68.6%) than female students (60.9%) (χ^2^ = 9.197, *p* = 0.002, fi = 0.061) (Table 3). Normal weight students were significantly more frequently engaged in some kind of regular PA (63.6%), while obese students most often were not regularly physically active (45.6%), in relation to the students with a different nutritional status (χ^2^ = 10.742, *p* = 0.013, fi = 0.066). Above-average household income medical students engaged in significantly more regular PA (69.0%), compared to students whose household income was below average (59.1%). The difference was statistically significant (χ^2^ = 19.168, *p* < 0.001, fi = 0.088). There was no significant difference between the percentage of students who engaged in some form of PA and students who did not engage regularly in PA, in relation to the year of study (χ^2^ = 0.511, *p* = 0.475, fi = 0.014), alcohol consumption (χ^2^ = 1.253, *p* = 0.869, fi = 0.023), type of settlement (χ^2^ = 1.339, *p* = 0.247, fi = 0.023), and student smoking status (χ^2^ = 1.954, *p* = 0.162, fi = 0.028).

### 3.1. Motives for Regular Physical Activity among Medical Students

Taking whole sample into consideration, regardless of other independent variables, the motives for regular PA of medical students were, in order of frequency: to feel better (52.7%), to reduce stress (38.6%), to look better (38.6%), the desire to lose weight (18.6%), and to control chronic disease (5.5%) (not shown in tables).

The statistically significant difference in the percentage was determined in relation to the attended faculty, for the motives for regular PA: to feel better (χ^2^ = 64.45, *p* < 0.001, fi = 0.162), stress reduction (χ^2^ = 82.022, *p* < 0.001, fi = 0.183), and to look better (χ^2^ = 47.471, *p* < 0.001, fi = 0.139) (Table 4).

The motives for regular PA—to feel better, stress reduction, and the desire to look better, was given by the students of the Faculty of Medicine of the University of Zenica, Bosnia and Herzegovina, in the largest percentage (74.6%, 61.0%, and 51.2%, respectively), compared to the other medical faculties (Table 4). Students from the Faculty of Pharmacy and Biochemistry of the University of Zagreb, Republic of Croatia, stated the motives for engaging in regular PA—to feel better and stress reduction in the lowest percentage (41.3%, and 30.3%, respectively), and the students from the Faculty of Pharmacy of the University of Belgrade, Republic of Serbia stated the desire to look better (29.0%), had the lowest percentage, in comparison to other medical faculties.

No statistically significant difference was found in the frequency of the responses from the medical students from the different faculties, for the motives for regular PA—desire to lose weight (χ^2^ = 20.92, *p* = 0.075, fi = 0.092) and to control chronic disease (χ^2^ = 14.277, *p* = 0.355, fi = 0.076) (Table 4).

To feel better, as a motive for regular PA, was more frequently stated by female students, compared to male students (χ^2^ = 11.067, *p* = 0.001, fi = 0.067), fourth-sixth year students vs. first-third year students (χ^2^ = 21.322, *p* < 0.008, fi = 0.093), normal weight vs. overweight, obese or underweight (χ^2^ = 6.98, *p* < 0.001, fi = 0.053), students who drink alcohol vs. do not drink alcohol (χ^2^ = 15.915, *p* = 0.003, fi = 0.125), had a household income above average vs. average and below average (χ^2^ = 38.578, *p* < 0.001, fi = 0.125), and there were non-smokers, in comparison to smokers (χ^2^ = 6.082, *p* = 0.014, fi = 0.05) (Table 5). There was no statistically significant difference in the frequency of the stated motive to feel better, for students in relation to the type of settlements (χ^2^ = 2.253, *p* = 0.133, fi = 0.03).

Stress reduction was a significantly more frequently stated motive by female (χ^2^ = 14.728, *p* < 0.001, fi = 0.078), underweight students (χ^2^ = 13.372, *p* = 0.004, fi = 0.074), who had a household income above average (χ^2^ = 16.22, *p* < 0.001, fi = 0.081), and were non-smokers (χ^2^ = 8.842, *p* = 0.003, fi = 0.06) (Table 5). There was no statistically significant difference in the frequency of the stated motives to reduce stress, for students in different years of study (χ^2^ = 2.788, *p* = 0.095, fi = 0.034), alcohol consumption (χ^2^ = 2.126, *p* = 0.713, fi = 0.029), and who were coming from different types of settlements (χ^2^ = 0.18, *p* = 0.672, fi = 0.009).

To look better, was significantly more often stated by female students (χ^2^ = 15.572, *p* < 0.001, fi = 0.08), normal weight (χ^2^ = 18.888, *p* < 0.001, fi = 0.088), those whose household income was above average (χ^2^ = 38.578, *p* < 0.001, fi = 0.125), and did not smoke (χ^2^ = 7.692, *p* = 0.006, fi = 0.056) (Table 5). There was no statistically significant difference in the frequency of the stated motive to look better for students in different years of study (χ^2^ = 2.23, *p* = 0.135, fi = 0.03), alcohol consumption (χ^2^ = 8.86, *p* = 0.065, fi = 0.06), and who were coming from different types of settlements (χ^2^ = 1.425, *p* = 0.233, fi = 0.024).

The desire to lose weight was significantly more frequently stated by overweight and obese students, compared to normal weight and underweight students (χ^2^ = 85.805, *p* < 0.001, fi = 0.187), and students whose household income was above average (χ^2^ = 12.529, *p* = 0.002, fi = 0.071) (Table 5). There was no statistically significant difference in the frequency of the stated motive for the desire to lose weight, for gender (χ^2^ = 0.335, *p* = 0.563, fi = 0.012), students in different years of study (χ^2^ = 1.935, *p* = 0.164, fi = 0.028), alcohol consumption (χ^2^ = 4.862, *p* = 0.302, fi = 0.045), who were coming from different types of settlements (χ^2^ = 0.147, *p* = 0.702, fi = 0.008), and smoking status (χ^2^ = 0.041, *p* = 0.840, fi = 0.84).

There was no statistically significant difference in the frequency for motive to control chronic disease, according to gender (χ^2^ = 0.003, *p* = 0.956, fi = 0.001), students in different years of study (χ^2^ = 0.921, *p* = 0.471, fi = 0.015), BMI (χ^2^ = 0.858, *p* = 0.836, fi = 0.019), alcohol consumption (χ^2^ = 4.682, *p* = 0.321, fi = 0.044), household income (χ^2^ = 0.761, *p* = 0.684, fi = 0.018), who are coming from different settlements (χ^2^ = 0.129, *p* = 0.720, fi = 0.007), and smoking status (χ^2^ = 0.722, *p* = 0.395, fi = 0.017) (Table 5).

### 3.2. Barriers to Regular Physical Activity among Medical Students

Taking the whole sample into consideration, regardless of other independent variables, faculty obligations were the most common barrier for regular PA among medical students (59.3%), followed by other barriers (14.2%), financial situation (8.6%), current life situation (8.4%), and health condition (2.8%) (not shown in tables).

The difference in percentage of the barriers for regular PA was determined in relation to the attended faculty and were statistically significant for: faculty obligations (χ^2^ = 46.876, *p* < 0.001, fi = 0.138), current life situation (χ^2^ = 37.353, *p* < 0.001, fi = 0.123), financial situation (χ^2^ = 28.106, *p* = 0.009, fi = 0.107), and other barriers (χ^2^ = 38.855, *p* < 0.001, fi = 0.107) (Table 6).

Obligations at the faculty was the most frequently given answer by students from the Faculty of Pharmacy of the University of Sarajevo, Bosnia and Herzegovina (76.9%), while from students from the Faculty of Pharmacy of the University of Belgrade, Republic of Serbia, faculty obligations was the lowest (44.4%), compared to other medical faculties (Table 6).

The highest percentage of students who stated their current life sitation as a barrier for regular PA, was from the Faculty of Medicine of the University of Zenica, Bosnia and Herzegovina (14.6%), and the lowest was from the Faculty of Health Studies of the University of Mostar, Bosnia and Herzegovina (1.9%) (Table 6).

The financial situation was mostly stated as the barrier by students from the Faculty of Pharmacy of the University of Sarajevo, Bosnia and Herzegovina (14.9%), and it was the least stated reason by those from the Faculty of Medicine of the University “St. Cyril and Methodius” Skopje, Republic of North Macedonia (2.2%) (Table 6).

Students of the Faculty of Medicine of the University of Rijeka, Republic of Croatia, stated other barriers, which fell in the highest percentage (21.9%), and students of the Faculty of Medicine of the University “St. Cyril and Methodius” Skopje, Republic of North Macedonia, stated other barriers, which fell in the lowest (8.4%) (Table 6).

No statistically significant difference was found in the frequency of health conditions, as a barrier for regular PA, in relation to the attended faculty (χ^2^ = 14.168, *p* = 0.362, fi = 0.076) (Table 6).

Faculty obligations as a barriers for regular PA was significantly more frequently stated by female, compared to male students (χ^2^ = 9.252, *p* = 0.002, fi = 0.061) (Table 7). There was no statistically significant difference in the frequency of the stated faculty obligations as a barrier, for students in a different year of study (χ^2^ = 0.068, *p* = 0.795, fi = 0.005), BMI (χ^2^ = 3.908, *p* = 0.272, fi = 0.04), alcohol consumption (χ^2^ = 6.14, *p* = 0.189, fi = 0.05), household income (χ^2^ = 5.172, *p* = 0.075, fi = 0.046), type of settlements (χ^2^ = 3.502, *p* = 0.061, fi = 0.038), and smoking status (χ^2^ = 0.888, *p* = 0.320, fi = 0.02).

The current life situation was significantly more frequently stated by male students (χ^2^ = 6.589, *p* = 0.010, fi = 0.052), who were overweight (χ^2^ = 13.521, *p* = 0.004, fi = 0.074), and whose household income was below average (χ^2^ = 42.49, *p* < 0.001, fi = 0.132) (Table 7). No significant difference in the frequency of stated current life situation, as a barrier for students, was determined for different years of study (χ^2^ = 2.375, *p* = 0.123, fi = 0.031), alcohol consumption (χ^2^ = 3.16, *p* = 0.531, fi = 0.036), who came from different types of settlements (χ^2^ = 0.019, *p* = 0.890, fi = 0.003) and smoking status (χ^2^ = 1.03, *p* = 0.310, fi = 0.02).

Financial situation was significantly more often stated by students who had a household income below average, compared to those with an average and above average household income (χ^2^ = 136.237, *p* < 0.001, fi = 0.236), and were smokers (χ^2^ = 9.178, *p* = 0.002, fi = 0.061) (Table 7). There was no statistically significant difference in the frequency of stated financial situation barrier, for gender (χ^2^ = 0.091, *p* = 0.763, fi = 0.006), different years of study (χ^2^ = 1.874, *p* = 0.171, fi = 0.028), BMI (χ^2^ = 2.036, *p* = 0.565, fi = 0.029), alcohol consumption (χ^2^ = 1.115, *p* = 0.892, fi = 0.021), and types of settlements (χ^2^ = 0.601, *p* = 0.438, fi = 0.016).

No statistically significant difference in the frequency of the stated health condition barrier was determined, in relation to gender (χ^2^ = 0.537, *p* = 0.464, fi = 0.021), year of study (χ^2^ = 2.763, *p* = 0.587, fi = 0.034), BMI (χ^2^ = 6.709, *p* = 0.082, fi = 0.052), alcohol consumption (χ^2^ = 2.584, *p* = 0.630, fi = 0.032), household income (χ^2^ = 1.087, *p* = 0.581, fi = 0.021), type of settlement (χ^2^ = 0.04, *p* = 0.842, fi = 0.004), and smoking status (χ^2^ = 1.978, *p* = 0.160, fi = 0.028).

Other barriers for regular PA were significantly more frequently stated by overweight and obese students (χ^2^ = 10.627, *p* = 0.014, fi = 0.066), and students who had an average household income, compared to those whose income was above average and below average (χ^2^ = 6.863, *p* = 0.032, fi = 0.053), who lived in rural settlements (χ^2^ = 15.638, *p* < 0.001, fi = 0.081), and were smokers (χ^2^ = 5.774, *p* = 0.016, fi = 0.049) (Table 7). There was no statistically significant difference in the frequency of other barriers, for gender (χ^2^ = 1.127, *p* = 0.288, fi = 0.021), year of study (χ^2^ = 2.763, *p* = 0.096, fi = 0.034), and alcohol consumption (χ^2^ = 0.763, *p* = 0.943, fi = 0.018).

### 3.3. Motives and Barriers for Regular Physical Activity as Predictors of Engagement in Physical Activity among Medical Students

The model of the univariate MNLR analysis showed that all motives and barriers for regular PA were significantly associated with the medical students’ PA (Table 8). To feel better was the highest positive predictor among students engaging in regular PA (OR: 15.204, 95% CI: 12.335–18.741), while the highest negative predictor was a health condition (OR: 0.579, 95% CI: 0.359–0.935).

The model of the multivariate MNLR was created with the aim of controlling the independent variables for their mutual influence. To feel better remained highest positive predictor of students engaging in regular PA (OR: 7.859, 95% CI: 6.221–9.929), while the highest negative predictor was the financial situation (OR: 0.528, 95% CI: 0.385–0.725) (Table 8).

Spearman’s Rank-Order correlation analysis showed a statistically significant positive moderate correlation between the motives to feel better and stress reduction, the desire to look better and to lose weight (Table 9). The desire to look better correlated moderately with a desire to lose weight. Other correlations between motives for regular PA were significant, but small.

A small, statistically significant, and positive correlation was determined between the barriers concerning the current life situation and financial situation. Other motives correlated negatively with faculty obligations. Other correlations between the barriers, although significant, were very small (Table 10).

## 4. Discussion

Our study shows that 62.3% of medical students engaged in some kind of regular PA. The male gender, normal weight, with an above average household income was positively associated with students’ PA. Similar to a study in Martinović et al. [23] conducted in Split, Republic of Croatia, where more than half of biomedical students engage in some kind of regular PA, the impact of greater knowledge about healthy behavior for maintaining good health and preventing disease, was emphasized as the main factor that impacts PA [23]. Pavičić-Žeželj et al. [24], in a study conducted in Rijeka (Republic of Croatia), and Ćatović and Halilović [25], in a study conducted in Sarajevo (Bosnia and Herzegovina), among medical students, indicate that normal weight is highly associated with the regular engagement in PA, which was not statistically significant in our paper. The same studies indicate that male students engaged in some kind of PA more often and had higher levels of PA, compared to female students [25]. Different studies report that low levels of PA are associated with a low household income [26], while others associate it with high physical inactivity [27]. A study by Grujičić et al. [9], on medical students from the Western Balkans, on the influence of different factors on daily PA levels, states that students who have higher daily PA levels, are more likely to be normal weight males whose household income is above average.

### 4.1. Motives for Regular Physical Activity among Medical Students

This is the first known study to examine the motives for regular physical activity among medical students from the Western Balkans. Our results imply that the motives in this research were in the order of frequency: to feel better, to reduce stress, to look better, to lose weight, and to control chronic disease. The results of our research show that motives to feel better, to reduce stress, and to look better, significantly differed in the frequency among the different medical faculties. Similarly, Lustyk et al. [28], in a study conducted on college medical students from the Pacific Northwest, indicate that a linear combination of the three motives (for fitness and health reasons, to control weight, shape, and appearance, and to improve mood, have fun, and socialize) accounted for 8% of the variance of the quality of life derived from PA [28]. According to the results of the study by Roberts et al. [29], which was conducted among undergraduate students from the northwest of England, health-related motives, such as positive health and weight management, are stated as the primary motivation for doing PA in one’s free time. A study by Dias et al. [30] indicates that competency/mastery factors and intellectual factors are the prime motivational factors that encouraged Indian college students to become involved in PA. Tao et al. [31] indicate that the autonomous motivation, such as improving one’s own health, is the strongest motive for Chinese college students to engage in PA.

Compared to other college students, medical students generally have a higher consciousness of health [23,32]. It has been indicated that medical students report higher PA levels with a high autonomous motivation (personal reasons for health improvement vs. educational programs) than age-matched peers in the general population [33], which is consistent with the high motives of medical students in this research. Laishram et al. [34] also conducted a study on the motives of medical students from India, regarding PA, and showed that the intrinsic and motives identified above are the highest two motives for medical students to participate in PA, compared to any external regulation (requirements during formal education). Consistent with our results, research among health sciences students from Poland indicates that the pleasure of PA is the most important motivation for undertaking it, followed by strong motivating factors, including mental regeneration, maintaining health, building strength and endurance, appearance, and avoiding ill health [35]. In our research, to feel better and to control chronic disease were the highest and lowest motives of regular PA, separately, for all faculties, which could suggest that there is a strong internal drive that indirectly reflects the good health condition and the relatively low prevalence of chronic diseases, among medical students. A positive moderate correlation between motives to feel better and stress reduction, the desire to look better and to lose weight, emphasize the importance of the internal motivation for maintaining regular PA.

Our study shows that female students more often engaged in regular PA to feel better, reduce stress, and look better, compared to male students. Furthermore, the results of our study indicate that although male students engage in PA more frequently than the female students stated, their motives for regular PA were in a significantly higher percentage than the male students. This result indicates that males and females have distinct motives for participation in PA at different levels. Furthermore, many studies on the motivation for participating in PA, points to significant gender-related disparities [29,34,36,37,38]. Romero-Blanco et al. [37], in a study conducted in Spain, found that male adults were more motivated than females by affiliation and challenge, whereas female adults were motivated more than males were, by a better appearance. Similar to these results, a study by Molanorouzi et al. [38] conducted among adults from Malaysia, shows that males have a higher motivation for mastery and competition/ego, whereas females have a higher motivation for appearance and physical condition [38]. In contrast with our results, the research from Malaysia suggested that there are no significance differences in the motives for taking part in PA, between the genders, among student-athletes [39]. Vanlalduhsaki et al. [34] show that there is a statistically significant higher intrinsic and identified motives for male medical students, compared to female medical students, from India.

The results of our study show that more advanced medical students have a significantly higher motive to feel better, with regard to PA, than less advanced students. Similar to our results, Roberts et al. [29], Chen et al. [40], and Martinez-Lacoba et al. [41] indicate that students above the age of 23 are more motivated than students in lower age groups (i.e., for stress, revitalization, and avoidance of ill health). The authors emphasize that it is possible that as college students grow older, they become more conscious of the value of exercise and good health [29,40,41]. Moreover, Roberts et al. [29], Chen et al. [40], and Martinez-Lacoba et al. [41] point out that, as a university students approach the end of their studies, their lifestyle management possibly improves. In contrast with our results, Egli et al. [36] reports that students under the age of 20 are more motivated for reasons connected to their health.

The obtained results of our research show that the motives of PA among medical students, had a significant connection with the BMI. Obese students showed significantly less stated motives to feel better, reduce stress, and to look better, but more often stated their motive to lose weight, compared to non-obese students. Baillot et al. [18] found that weight management is the key PA motive among people with obesity. In addition, in a study conducted by Rao et al. [32] among undergraduate medical students in India, the results show that the proportion of medical students who participate in PA increased, as the BMI increased [32]. The Congruence of such findings was also reported by Banerjee et al. [42], in a study conducted in India. Overall, those results suggest that the BMI is significantly related to different motivations for students who indulge in various types of PA [42]. In contrast with the results of our study, Pavičić-Žeželj et al. [24], in a study conducted in Croatia, point out that normal weight medical students more often engage in PA for maintaining a good health, which is not statistically significant and the least frequent motive for regular PA, in our research.

The result of our study indicates that students whose household income was above average, more often engaged in PA with motives stated were to feel better, to reduce stress, look better, and to lose weight, compared to the average and below average students. Dąbrowska-Galas et al. [43], in a study conducted in Poland and Grujičić et al. [9], in a study conducted in Serbia, indicate that the fact that the universities of the Western Balkans do not have programs that would enable free sports centers for all students, is the main reason why students from family with an above average household income are more often engaged in regular PA. Higher household incomes bring more chances to access more and better sports infrastructure, such as swimming pools, and other kinds of developed fitness equipment [9,43]. Berger et al. [44], in a study conducted on students from the Višegrad countries, indicate that a better economic situation in the Višegrad countries provides more free time and opportunities for participation in regular PA. The same findings were also reported in many other societies and groups [43,44,45,46]. More opportunity for practicing regular PA brings more pleasure and naturally more pleasure, in turn, increases the motivation to do engage in more regular PA [43,44,45,46]. Therefore, it is not difficult to understand that in our research, students with an above-average household income showed statistically significant higher motives, including all motives, except to control chronic disease.

Our research indicates that students who are non-smokers stated the desire to feel better, reduce stress, and to look better, as motives for regular PA, more often than smokers. Smoking has been a significant public health problem among adolescents and college students [47,48]. Among global medical students, it is estimated that one in five students smoke [47,48]. The result of a study conducted in the Western Balkans, on smoking among medical students, shows that the percentage of smokers is significantly lower than that of non-smokers [48]. In parallel, the results of our study show that non-smokers had significantly higher motives (to feel better, reduce stress, and to look better), in regular PA. Smoking has been shown to have both short-term and long-term consequences on PA, including a poorer endurance, decreased physical performance, increased risk of injury and complications, etc. [49]. In a study conducted in Tehran, Heydari et al. [50] found that smokers have a higher odds ratio of 4.88, for having an unsatisfactory level of PA, in relation to non-smokers [50]. Heydari et al. [50] point out that the negative effects of smoking on PA possibly further contributes to lower motives in PA [50]. However, a study of the interaction effects of life satisfaction and PA on smoking initiation among students,, indicates that lower levels of life satisfaction are associated with a greater likelihood of smoking initiation [51]. Thus, it is reasonable to believe that smokers, in our research, feel better when they smoke and that cigarette reduces stress for them, or they do not pay much attention to their looks because they already smoke [51]. Although, in contrast with our results, this research by Torchyan et al. [51] shows that high levels of PA increased the chance of smoking initiation among male students with high levels of life satisfaction [51]. Therefore, the detailed potential mechanism under this phenomenon should be further investigated.

More interestingly, the results of our research show that, compared with students who do not drink, students who drink alcohol, significantly recognize the need to feel better as a motive for regular PA. Sjödin et al. [52] indicate that the enhancement of mood and dealing with stress are the second strongest drinking motive, behind social motives among adolescents in Sweden. Among Korean students, Kim et al. [53] also show that among the various motives for drinking, the enhancement motives are the second most common, behind the social motives. Enhancement motives involve drinking to increase the positive affect because of the active dopamine system caused by alcohol [54,55]. Furthermore, regular PA makes people feel better, due to the endorphins [56]. The two systems have a similar role in developing behavioral habits, which may indirectly explain the significant motive for the need to feel better, in PA, for daily alcohol drinker students, compared to students who do not drink alcohol [54,55,56].

The result of our research shows that there was no statistically significant difference in the frequency of all motives between students from rural and urban settlements. Similarly to our results, a study by Moreno-Llamas et al. [57], that investigated the urban-rural differences in the trajectories of PA in Europe, from 2002 to 2017, showed that there was no significant difference of daily regular PA between students from rural and urban areas. The same study indicated that a trend of inactivity prevalence increased in both rural and urban environments, across time [57]. The same study also showed that the type of settlement does not affect the cognitive component of engaging in PA for medical students, including the frequency and motives for PA [57].

### 4.2. Barriers for Regular Physical Activity among Medical Students

The results of our research indicate that faculty obligations were the strongest barrier for medical students, concerning regular PA. Due to the particularity of the major of medicine, the course and practice trainings for medical students can be physically and emotionally demanding [58]. Thus, the faculty obligations facing medical students, including academic requirements, the nature of the contents, the heavy workload, and the exams, are larger than other university students [59,60], which could lead to the difficulty in finding time to participate in regular PA. Similar to our study, the results of a study by Likus et al. [61], show that workload is the biggest barrier to PA for Polish medical students. Furthermore, a significant amount of evidence has also demonstrated that, among university students, the lack of time, due to faculty obligations is the most common barrier to PA [19,37,62]. In a recent systematic review of various barriers to university students’ PA, 22 studies with university students were included [63]. The authors have also concluded that a lack of time, due to faculty obligations, was the most cited barrier to PA, among university students [63].

Current life situation, financial situation, and other barriers, are leading barriers among the medical students in our research after faculty obligations. Similar to our study, Ferreira-Silva et al. [63] emphasize that personal circumstances are also barriers to regular PA. The same study shows that the barriers to regular PA in the psychological, emotional, and cognitive categories are similar in students from all parts of the world. In our research, only the current health condition was not recognized as a significant barrier by the students, which also indirectly reflects the possible good health condition among students, also consistent with lowest motive (to control chronic disease) for regular PA. Furthermore, the statistical significance in the frequency of the barriers (except health conditions), differed between medical faculties. More interestingly, in our study, the current life situation was significantly positively correlated with the financial situation, while faculty obligations was significantly negatively correlated with other barriers, indicating the relationship between the various socioeconomic and individual factors.

Contrary to our results, Hoare et al. [64] indicate that there is no gender difference in the perceived barriers to PA, between Australian females and males. In our study, female students more frequently recognized faculty obligations as a regular PA barrier, while they less frequently recognized their current life situation as a regular PA barrier, than male students. It is possible that female medical students might need more time for faculty obligations, but they hold stronger the ability or awareness of facing and handling current life situations, than men do. Similar to our research, a study by Shin at al. [65] also suggests that there are systematic differences between PA barriers related to the variables, including gender. In a study in Florence, Italy, Rosselli et al. [66] found that the number of perceived barriers to PA was higher among females than among males.

Our study shows that medical students whose household income was below average stated their current life situation and financial situation as a reason for irregular PA, more often, compared to average and above average medical students. A higher household income is associated with higher motives for regular PA because of more opportunities and more access to better sports infrastructure [43,44]. Equally, it is easy to understand that in this research, the financial situation was a significant barrier for PA and the above-average household income was significantly negatively associated with the barriers (current life situation and financial situation).

The result of our research shows that overweight and obese students stated their current life situation as a reason for not engaging in regular PA, which is almost two times more than the students with a different nutritional status. The results suggest that overweight and obesity might lead to a negative influence on students’ current life situation, such as special events, day-to-day or global circumstances, or personal circumstances, that affect the person or their family or friends (e.g., illness or a situation that requires care) [67]. Further, it is supposed that the negative impacts caused by a previous unhealthy lifestyle, can have a stimulating effect in engaging in regular PA, among medical students [67].

The obtained results of our research indicate that smokers stated their financial situation more often than non-smokers, as a barrier for PA. A study carried out by Wamamili [68], investigated the change in smoking intentions among university students in New Zealand, following the cigarette price increase. According to the results of the study conducted in New Zealand, the proportion of students who would smoke declined substantially, as the price of cigarettes increased by NZD 5, 10, and 15 [68]. The authors point out that smoking accounts as a considerable financial expense for university students, therefore their financial situation is more often stated as a PA barrier by students who smoke [68].

Our study has several limitations. Conducted in the form of a cross-sectional study, only the currently observed situation was possible to be analyzed, and tracking changes over and the cause-effect relationship, could not be observed [69]. The reliability of the gathered data was unable to be determined, despite emphasizing on anonymity and confidentiality, the respondents could frequently be dishonest in giving answers while using an online, anonymously filled-in, self-administered questionnaire [69]. Finally, medical faculties were selected, using the convenience sampling method and were not randomly selected, so the obtained results cannot be generalized to all Western Balkan medical students. Although we were unable to gather the exact numbers (response rate) of the enrolled students at the period of the study conduction, the percentage of students from all of individual faculties exceeds a representative 10% [9,48].

## 5. Conclusions

The most reported motive among medical students for regular PA is to feel better, followed by stress reduction, to look better, the desire to lose weight, and to control chronic disease. PA improvement motives are more frequently associated with the female gender, more advanced years of study, normal weight, an above average household income, and a non-smoking status. Faculty obligations are the most common barrier for regular PA among medical students, followed by other barriers, such as their financial situation, current life situation, and health condition. Barriers are more frequently reported by male students who are overweight or obese, who have a below average income and who are smokers.

It is necessary for the public health authorities to examine and take into account the perceived motives and barriers, when forming activities and policies that aim at increasing the level of PA, in order to enhance the health of the student population.

## Figures and Tables

**Table 1:** Sample structure.

Country	*n*	%	Faculty	*n*	%
Republic of Slovenia	218	8.9	Faculty of Medicine of the University of Ljubljana	218	8.9
Republic of Croatia	417	17.0	Faculty of Pharmacy and Biochemistry of the University of Zagreb	271	11.0
			Faculty of Medicine of the University of Rijeka	146	6.0
Bosnia and Herzegovina	878	35.8	Faculty of Medicine of the University of Sarajevo	128	5.2
			Faculty of Pharmacy of the University of Sarajevo	121	4.9
			Faculty of Health Studies of the University of Sarajevo	208	8.6
			Faculty of Medicine of the University of Zenica	205	8.4
			Faculty of Pharmacy of the University of Mostar	110	4.5
			Faculty of Health Studies of the University of Mostar	106	4.4
Republic of North Macedonia	306	12.5	Faculty of Medicine of the University “St. Cyril and Methodius” Skopje	179	7.4
			Faculty of Pharmacy of the University “St. Cyril and Methodius” Skopje	127	5.2
Republic of Serbia	633	25.8	Faculty of Pharmacy of the University of Belgrade	124	5.1
			Faculty of Medicine Novi Sad of the University of Novi Sad	370	15.3
			Faculty of Pharmacy of the University of Business Academy Novi Sad	139	5.7

**Table 2:** Distribution of the medical students (*n* = 2452), according to regular daily physical activity, in relation to the attended faculty.

Country	Faculty	Regular Daily Physical Activity				*p* *
		No Regular Physical Activity		Engaging in Some Kind of Regular Physical Activity		
		*n*	%	*n*	%	
Republic of Slovenia	Faculty of Medicine of the University of Ljubljana	104	47.7	114	52.3	<0.001
Republic of Croatia	Faculty of Pharmacy and Biochemistry of the University of Zagreb	138	50.9	133	49.1	
	Faculty of Medicine of the University of Rijeka	61	41.8	85	58.2	
Bosnia and Herzegovina	Faculty of Medicine of the University of Sarajevo	54	42.2	74	57.8	
	Faculty of Pharmacy of the University of Sarajevo	57	47.1	64	52.9	
	Faculty of Health Studies of the University of Sarajevo	62	29.8	146	70.2	
	Faculty of Medicine of the University of Zenica	50	24.4	155	75.6	
	Faculty of Pharmacy of the University of Mostar	25	22.7	85	77.3	
	Faculty of Health Studies of the University of Mostar	38	35.8	68	64.2	
Republic of North Macedonia	Faculty of Medicine of the University “St. Cyril and Methodius” Skopje	58	32.4	121	67.6	
	Faculty of Pharmacy of the University “St. Cyril and Methodius” Skopje	39	30.7	88	69.3	
Republic of Serbia	Faculty of Pharmacy of the University of Belgrade	56	45.2	68	54.8	
	Faculty of Medicine Novi Sad of the University of Novi Sad	140	37.8	230	62.2	
	Faculty of Pharmacy of the University of Business Academy Novi Sad	43	30.9	96	69.1	

* *p* value calculated by using the χ^2^ test for the categorical variables. Significant at *p* < 0.05.

**Table 3:** Regular daily physical activity of medical students, in relation to the independent variables.

Variables		Regular Daily Physical Activity				*p* *
		No Regular Physical Activity		Engaging in Some Kind of Regular Physical Activity		
		*n*	%	*n*	%	
Gender	Male	137	31.4	300	68.6	0.002
	Female	788	39.1	1227	60.9	
Year of study	1–3	626	38.2	1012	61.8	0.475
	4–6	299	36.7	515	63.3	
BMI	Underweight	73	42.9	97	57.1	0.013
	Normal weight	703	36.4	1227	63.6	
	Overweight	118	40.0	177	60.0	
	Obese	31	54.4	26	45.6	
Alcohol consumption	I do not drink alcohol	318	38.7	504	61.3	0.869
	Occasionally	409	37.2	691	62.8	
	On weekends	158	36.5	275	63.5	
	Several times a week	35	41.2	50	58.8	
	Daily	5	41.7	7	58.3	
Household income	Below average	110	40.9	159	59.1	<0.001
	Average	593	40.4	875	59.6	
	Above average	222	31.0	493	69.0	
Type of settlement	Rural	272	36.0	483	64.0	0.247
	Urban	653	38.5	1044	61.5	
Smoking status	Non-smoker	707	37.0	1204	63.0	0.162
	Smoker	218	40.3	323	59.7	

* *p* value calculated by using the χ^2^ test for the categorical variables. Significant at *p* < 0.05.

**Table 4:** Motives for regular physical activity among medical students, in relation to the attended faculty (%).

Country	Faculty	Motives for Regular Physical Activity				
		To Feel Better	Stress Reduction	To Look Better	The Desire to Lose Weight	To Control Chronic Disease
Republic of Slovenia	Faculty of Medicine of the University of Ljubljana	49.1	31.7	30.7	16.5	6.4
Republic of Croatia	Faculty of Pharmacy and Biochemistry of the University of Zagreb	41.3	30.3	33.2	16.2	3.7
	Faculty of Medicine of the University of Rijeka	53.4	32.2	38.4	15.8	6.8
Bosnia and Herzegovina	Faculty of Medicine of the University of Sarajevo	46.9	32.0	32.8	18.0	7.8
	Faculty of Pharmacy of the University of Sarajevo	47.9	35.5	30.6	19.8	4.1
	Faculty of Health Studies of the University of Sarajevo	53.4	35.1	40.9	23.1	7.7
	Faculty of Medicine of the University of Zenica	74.6	61.0	51.2	25.4	7.3
	Faculty of Pharmacy of the University of Mostar	53.6	39.1	33.6	16.4	3.6
	Faculty of Health Studies of the University of Mostar	55.7	33.0	44.3	14.2	2.8
Republic of North Macedonia	Faculty of Medicine of the University “St. Cyril and Methodius” Skopje	57.0	50.8	48.6	25.1	6.1
	Faculty of Pharmacy of the University “St. Cyril and Methodius” Skopje	52.0	41.7	41.7	15.7	3.9
Republic of Serbia	Faculty of Pharmacy of the University of Belgrade	43.5	31.5	29.0	16.9	2.4
	Faculty of Medicine Novi Sad of the University of Novi Sad	54.9	42.7	42.4	18.1	5.1
	Faculty of Pharmacy of the University of Business Academy Novi Sad	51.1	33.8	33.8	14.4	7.9
*p* *		<0.001	<0.001	<0.001	0.075	0.335

* *p* value calculated by using the χ^2^ test for the categorical variables. Significant at *p* < 0.05. Note: The question was designed as a multiple-choice, where students could have chosen one or more offered answers.

**Table 5:** Motives for regular physical activity among medical students, according to gender, year of study, body mass index (BMI), alcohol consumption, household income, type of settlement, and student smoking status.

Variables		Motives for Regular Physical Activity									
		To Feel Better		Stress Reduction		To Look Better		The Desire to Lose Weight		To Control Chronic Disease	
		%	p *	%	p *	%	p *	%	p *	%	p *
Gender	Male	51.2	0.001	36.8	<0.001	36.8	<0.001	18.8	0.563	5.6	0.956
	Female	60.0		46.7		46.9		17.6		5.5	
Year of study	1–3	50.9	0.008	37.4	0.095	37.5	0.135	17.8	0.164	5.3	0.471
	4–6	56.5		40.9		40.7		20.1		6.0	
BMI	Underweight	44.1	<0.001	40.0	0.004	30.6	<0.001	3.5	<0.001	6.5	0.836
	Normal weight	55.5		39.6		40.1		17.1		5.6	
	Overweight	45.1		34.9		38.0		35.3		5.1	
	Obese	24.6		17.5		15.8		28.1		3.5	
Alcohol consumption	I do not drink alcohol	47.1	0.003	36.7	0.713	34.7	0.065	17.3	0.302	5.6	0.321
	Occasionally	55.5		39.9		41.2		19.0		5.5	
	On weekends	55.4		38.3		39.0		21.0		6.0	
	Several times a week	56.5		40.0		41.2		12.9		2.4	
	Daily	58.3		41.7		33.3		25.0		16.7	
Household income	Below average	46.1	<0.001	39.4	<0.001	32.0	<0.001	20.8	0.002	6.7	0.684
	Average	49.3		35.6		35.2		16.3		5.4	
	Above average	62.4		44.5		48.0		22.4		5.5	
Type of settlement	Rural	50.5	0.133	39.2	0.672	36.8	0.233	18.1	0.702	5.3	0.720
	Urban	53.7		38.3		39.4		18.8		5.7	
Smoking status	Non-smoker	54.1	0.014	40.1	0.003	40.0	0.006	18.7	0.840	5.3	0.395
	Smoker	48.1		33.1		33.5		18.3		6.3	

* *p* value calculated by using the χ^2^ test for the categorical variables. Significant at *p* < 0.05. Note: The question was designed as a multiple-choice, where students could have chosen one or more offered answers.

**Table 6:** Barriers for regular physical activity among medical students, in relation to the attended faculty (%).

Country	Faculty	Barriers for Regular Physical Activity				
		Faculty Obligations	Current Life Situation	Financial Situation	Health Condition	Other Barriers
Republic of Slovenia	Faculty of Medicine of the University of Ljubljana	62.4	5.5	6.0	5.5	16.5
Republic of Croatia	Faculty of Pharmacy and Biochemistry of the University of Zagreb	66.8	10.3	10.3	3.3	10.7
	Faculty of Medicine of the University of Rijeka	56.8	9.6	13.0	2.1	21.9
Bosnia and Herzegovina	Faculty of Medicine of the University of Sarajevo	59.4	5.5	8.6	2.3	18.8
	Faculty of Pharmacy of the University of Sarajevo	76.9	12.4	14.9	0.8	9.9
	Faculty of Health Studies of the University of Sarajevo	56.7	9.1	9.6	2.9	15.4
	Faculty of Medicine of the University of Zenica	48.3	14.6	8.8	2.4	5.9
	Faculty of Pharmacy of the University of Mostar	58.2	9.1	7.3	2.7	11.8
	Faculty of Health Studies of the University of Mostar	61.3	1.9	7.5	1.9	15.1
Republic of North Macedonia	Faculty of Medicine of the University “St. Cyril and Methodius” Skopje	60.3	7.3	2.2	1.7	8.4
	Faculty of Pharmacy of the University “St. Cyril and Methodius” Skopje	55.9	8.7	3.9	3.9	15.7
Republic of Serbia	Faculty of Pharmacy of the University of Belgrade	44.4	13.7	12.1	4.8	21.0
	Faculty of Medicine Novi Sad of the University of Novi Sad	59.2	4.3	8.9	2.7	16.5
	Faculty of Pharmacy of the University of Business Academy Novi Sad	62.6	7.9	7.9	0.7	14.4
*p* *		<0.001	<0.001	0.009	0.362	<0.001

* *p* value calculated by using the χ^2^ test for the categorical variables. Significant at *p* < 0.05. Note: The question was designed as a multiple-choice, where students could have chosen one or more offered answers.

**Table 7:** Barriers for regular physical activity among medical students, according to gender, year of study, body mass index (BMI), alcohol consumption, household income, type of settlement, and student smoking status.

Variables		Barriers for Regular Physical Activity									
		Faculty Obligations		Current Life Situation		Financial Situation		Health Condition		Other Barriers	
		%	p *	%	p *	%	p *	%	p *	%	p *
Gender	Male	52.9	0.002	11.4	0.010	8.2	0.763	2.3	0.464	12.6	0.288
	Female	60.7		7.7		8.7		2.9		14.5	
Year of study	1–3	59.2	0.795	7.8	0.123	8.1	0.171	2.7	0.587	15.0	0.096
	4–6	59.7		9.6		9.7		3.1		12.5	
BMI	Underweight	66.5	0.272	7.1	0.004	10.0	0.565	3.5	0.082	11.8	0.014
	Normal weight	58.7		7.6		8.2		2.4		13.6	
	Overweight	59.3		13.6		10.2		4.1		17.3	
	Obese	59.6		12.3		10.5		7.0		26.3	
Alcohol consumption	I do not drink alcohol	62.5	0.189	8.5	0.531	8.6	0.892	3.5	0.630	13.9	0.943
	Occasionally	57.3		7.5		8.5		2.5		14.3	
	On weekends	59.4		10.2		8.3		2.5		15.0	
	Several times a week	56.5		9.4		9.4		2.4		12.9	
	Daily	50.0		8.3		16.7		0.0		8.3	
Household income	Below average	59.1	0.075	18.6	<0.001	25.7	<0.001	2.6	0.581	14.9	0.032
	Average	61.0		7.6		8.6		2.6		15.5	
	Above average	55.9		6.2		2.2		3.4		11.3	
Type of settlement	Rural	56.6	0.061	8.5	0.890	7.9	0.438	2.9	0.842	18.4	<0.001
	Urban	60.6		8.3		8.9		2.8		12.3	
Smoking status	Non-smoker	59.2	0.320	8.1	0.310	7.7	0.002	2.6	0.160	13.3	0.016
	Smoker	57.5		9.4		11.8		3.7		17.4	

* *p* value calculated by using the χ^2^ test for the categorical variables. Significant at *p* < 0.05. Note: The question was designed as a multiple-choice, where students could have chosen one or more offered answers.

**Table 8:** Association between the independent variables (motives and barriers for regular physical activity) and engagement in regular physical activity among medical students.

	Variables	Univariate Logistic Regression			Multivariate Logistic Regression		
		OR	95% CI for OR	*p*	OR	95% CI for OR	*p*
**Motives**	To feel better	15.204	12.335–18.741	<0.001	7.859	6.221–9.929	<0.001
	Stress reduction	8.202	6.592–10.205	<0.001	3.108	2.410–4.007	<0.001
	To look better	7.184	5.811–8.880	<0.001	1.808	1.379–2.371	<0.001
	To lose weight	2.879	2.250–3.683	<0.001	1.518	1.111–2.074	0.009
	To control chronic disease	4.220	2.552–6.978	<0.001	1.870	1.026–3.411	0.041
**Barriers**	Faculty obligations	0.301	0.251–0.361	<0.001	0.174	0.139–0.219	<0.001
	Current life situation	0.461	0.345–0.615	<0.001	0.408	0.292–0.569	<0.001
	Financial situation	0.416	0.313–0.554	<0.001	0.528	0.385–0.725	<0.001
	Health condition	0.579	0.359–0.935	0.025	0.434	0.256–0.737	0.002
	Other barriers	0.338	0.268–0.427	<0.001	0.122	0.091–0.164	<0.001

Abbreviations: OR—odds ratio; CI—confidence interval.

**Table 9:** Correlation between the motives for regular physical activity among medical students.

Correlation between the Motives for Regular Physical Activity	To Feel Better	Stress Reduction	To Look Better	To Lose Weight	To Control Chronic Disease
**To feel better**	1	0.475 **	0.524 **	0.184 **	0.122 **
**Stress reduction**	.	1	0.422 **	0.140 **	0.163 **
**To look better**	.	.	1	0.336 **	0.137 **
**To lose weight**	.	.	.	1	0.122 **
**To control chronic disease**	.	.	.	.	1

Level of significance for Spearman rank correlation coefficient—** *p* < 0.001.

**Table 10:** Correlation between the barriers for regular physical activity among medical students.

Correlation between the Barriers for Regular Physical Activity	Faculty Obligations	Current Life Situation	Financial Situation	Health Condition	Other Barriers
**Faculty obligations**	1	0.040 *	0.141 **	0.020	−0.253 **
**Current life situation**	.	1	0.249 **	−0.007	−0.089 **
**Financial situation**	.	.	1	−0.008	−0.096 **
**Health condition**	.	.	.	1	−0.062 **
**Other motives**	.	.	.	.	1

Level of significance for Spearman rank correlation coefficient—* *p* < 0.05, ** *p* < 0.001.

## Data Availability

The data presented in this study are available upon reasonable request from the corresponding author.
